# Reaction of quartz glass in lithium-containing alkaline solutions with or without Ca

**DOI:** 10.1098/rsos.180797

**Published:** 2018-09-12

**Authors:** Baofu Zhou, Zhongyang Mao, Min Deng

**Affiliations:** 1 College of Materials Science and Engineering, Nanjing Tech University, Nanjing 210009, People's Republic of China; 2 State Key Laboratory of Materials-Oriented Chemical Engineering, Nanjing 210009, People's Republic of China

**Keywords:** alkali–aggregate reaction, quartz glass, lithium silicate, calcium silicate hydrate

## Abstract

The exact role of lithium ions (Li^+^) in controlling alkali–silica reaction is still unclear. Thus, the effects of Li^+^ on the reaction between reactive silica (quartz glass) and hydroxyl in alkaline solution with or without Ca were investigated by quartz glass powder or slice immersion experiments. When quartz glass was immersed in lithium-containing alkaline solutions, only Li_2_SiO_3_ was produced with the absence of Ca, but Li_2_SiO_3_ and calcium silicate hydrate (CSH) were formed with the presence of Ca. The quartz glass slice immersion experiment indicated that the mass loss of quartz slices was less than 1% only when Ca was present in the lithium-containing alkaline solution. This was because a dense, low-porosity and strongly bonded production layer mainly composed of CSH and Li_2_SiO_3_ crystals was formed on the glass surface and served as a barrier against the diffusion of OH^−^ and alkali ions to the substrate glass.

## 1. Introduction

The use of lithium compounds to mitigate alkali–silica reaction (ASR) has been extensively investigated ever since the beneficial effects of lithium ions (Li^+^) on ASR-caused expansion were reported in 1951 [1]. However, the exact role of Li^+^ in controlling ASR is still unclear [2–5]. Several mechanisms have been proposed, including (i) enhancement in chemical stability of reactive silica exposed to pore solution [2,6–8], (ii) formation of less expansive Si–Li reaction products [9–12] and (iii) formation of physical barrier by insoluble Si–Li reaction products [8,9,13,14]. However, these mechanisms mostly have been determined by experiments with specific conditions and techniques, and do not apply to general situations. The three mechanisms are briefly discussed below.

For the first mechanism, the rate of silica dissolution is reportedly decelerated with the presence of lithium admixtures in systems, even though the pH of the pore solution remains essentially unchanged [2,7]. This observation implies that the presence of lithium ions somehow enhances the chemical stability of the silica, and in turn reduces the expansive gel amount and damage degree. The beneficial effects of LiNO_3_ in controlling ASR may be most reasonably explained by the strengthened chemical stability of reactive silica [2]. However, this mechanism only expounds the results when the pore solution contains Li^+^, but does not explain the exact nature in the enhancement of chemical stability.

For the second mechanism, the Li^+^ concentration in pore solution extracted from mortars would decrease with time while the Na^+^ and K^+^ concentrations remain more or less unchanged [2,9,15–17]. This phenomenon suggests that the Si–Li reaction is more preferential than the Na/K–silica reaction, and non-expansive lithium-bearing product may be formed instead of the classic expansive Na/Ca-containing ASR gel [10,12]. However, insufficient evidence supports the existence of non-expansive lithium-bearing products or helps to understand their structures or properties.

For the third mechanism, it has been postulated that insoluble Si–Li reaction products may be deposited on the surface of the reactive aggregate particles and act like a physical barrier against further attack to siloxane (Si–O–Si) groups by alkalis. The Si–Li complex formed with the presence of LiOH and opal is very insoluble and thus produces on the surface of the reactive silica particles an insoluble coating that effectively protects further participation in ASR [8]. Scanning electron microscopy (SEM) proves the formation of Si–Li crystals and Li-bearing ASR gel after glass discs (Vycor^™^) are immersed in a solution containing NaOH, LiNO_3_ and Ca(OH)_2_ [13,18]. Opal particles immersed in 1 mol l^−1^ LiOH solution saturated with Ca(OH)_2_ would be covered with a reaction product [14]. A study concerning the effects of Li^+^ on chemical sequence of ASR in a model reactant system as α-cristobalite to reactive silica suggests that the products should be mostly composed of silicon, lithium and calcium [19]. These studies indicate that the most probable mechanism explaining the beneficial ASR-controlling effect of Li^+^ might be the formation of a local physical barrier on the exposed surface of the reactive aggregate (i.e. surface imperfection, cracks and pores) by the products.

The key of the physical barrier mechanism is to understand the process and conditions of the formation, and the structure and composition of the physical barrier. Unfortunately, the structure and composition are hard to investigate especially in mortar or concrete, and even the energy-dispersive spectrum (EDS) cannot detect lithium when SEM/EDS was used. Furthermore, this physical barrier may be very thin on the aggregate–cement interface, which further complicates the research. Most previous studies cannot directly prove the existence of a physical barrier (or production layer), let alone understanding its composition, structure or the protective effect [2,20,21].

Maraghechi *et al*. [22] studied the effect of calcium on dissolution and precipitation reactions of different Pyrex glasses at high alkalinity and investigated the dissolution rate and products by SEM, transmission electron microscopy (TEM) and other analytical methods. This is an effective way to study the effect of Li^+^ on ASR with quartz glass as the reactive silica, because the reaction system is simple and the products can be easily distinguished from the reactants. The present study was aimed to further understand the ASR-controlling role of Li^+^ when quartz glass was used as the active aggregate. For this aim, the essential problem is to understand the products of the Li^+^ and quartz glass reaction in alkaline solution (pore solution of concrete) and how the product can inhibit or mitigate ASR. Specifically, this problem was studied by quartz glass powder or slice immersion experiments. LiNO_3_ was used as the source of Li^+^, as it outperforms other lithium compounds owing to its benign effect on concrete properties, neutrality and high solubility [9,16,18].

## 2. Material and methods

### 2.1. Material

To simplify the experimental system and facilitate the analysis of results, we used quartz glass, which has very high alkali activity [18,23], as the source of reactive silica. Tests with China Association for Engineering Construction Standardization (CECS) 48-1993 [24] show that the expansion rate of mortar with quartz glass is 1.2% after 6 h of autoclaving. Chemical analysis shows that the mass fraction of SiO_2_ in quartz glass is more than 99.9%. Quartz glass exists in two forms: quartz glass slice (QS) and quartz glass powder (QP). In this study, quartz glass slices (25 × 25 × 2 mm) were bought from Lianyungang, JiangSu province, China. Quartz glass powder was prepared by ball-milling the quartz glass slices into the size fraction of less than 160 µm.

The NaOH solution was prepared by dissolving the reagent grade NaOH with deionized water. The reagent grade LiNO_3_ and Ca(OH)_2_ were used as the sources of Li^+^ and Ca^2+^, respectively.

### 2.2. Mass-loss rate of quartz glass slices after immersion in different alkaline solutions

To examine how different alkaline solutions would influence the dissolution rate of quartz glass slices, we set up five systems:

1. *QS–Na system*: quartz glass slices immersed in 1 mol l^−1^ NaOH solution, without adding Ca(OH)_2_ or LiNO_3_.
2. *QS–Li system*: quartz glass slices immersed in 1 mol l^−1^ NaOH solution, with 1 mol l^−1^ LiNO_3_ added.
3. *QS–Ca system*: quartz glass slices immersed in 1 mol l^−1^ NaOH solution, under saturation with Ca(OH)_2_.
4. *QS–Li–Ca system*: quartz glass slices immersed in 1 mol l^−1^ NaOH solution, under saturation with Ca(OH)_2_ and 1 mol l^−1^ LiNO_3_.
5. In addition to the above four systems, to facilitate the in-depth analysis about the effect of Li^+^ concentration on mass-loss rate of QS in long time, we analysed the alkaline solutions containing different Li^+^ concentrations. Specifically, quartz glass slices were immersed in alkaline solutions with 1, 2 or 4 mol l^−1^ LiNO_3_ added at 80°C and immersed in alkaline solutions with 2 mol l^−1^ LiNO_3_ at 38°C for 200 days reaction (labelled as QS–1Li–Ca, QS–2Li–Ca, QS–4Li–Ca and QS–2Li–Ca-38 in table 1, respectively).

**Table 1.** Quartz glass slices immersion experiment.

system	samples	alkaline solution	Ca(OH)_2_ (g)	solution amount (ml)	temperature (°C)	reaction time (days)
1	QS–Na	1 mol l^−1^ NaOH	—	50	25, 38, 60, 80	120
2	QS–Li	1 mol l^−1^ NaOH + 1 mol l^−1^ LiNO_3_	—	50	25, 38, 60, 80	120
3	QS–Ca	1 mol l^−1^ NaOH	0.5	50	25, 38, 60, 80	120
4	QS–Li–Ca	1 mol l^−1^ NaOH + 1 mol l^−1^ LiNO_3_	0.5	50	25, 38, 60, 80	120
5	QS–1Li–Ca	1 mol l^−1^ NaOH + 1 mol l^−1^ LiNO_3_	0.5	50	80	200
	QS–2Li–Ca	2 mol l^−1^ NaOH + 1 mol l^−1^ LiNO_3_	0.5	50	80	200
	QS–4Li–Ca	4 mol l^−1^ NaOH + 1 mol l^−1^ LiNO_3_	0.5	50	80	200
	QS–2Li–Ca-38	2 mol l^−1^ NaOH + 1 mol l^−1^ LiNO_3_	0.5	50	38	200

Each slice was sealed in a polypropylene copolymer container containing 50 ml of different alkaline solutions, and thus, the glass surface area to solution volume ratio (SA/V) was 30 m^−1^, which ensured the reaction area to be same in all the experiments. The types of alkaline solutions and experimental temperatures are listed in table 1. Masses of glass slices in the first four systems were measured by an analytical balance after reaction for 7, 14, 28, 60, 90 and 120 days and exactly to the four decimal places, and after reaction for 200 days in the fifth system. The mass-loss rate of a quartz glass slice, *r*_t_, was calculated as follows:

2.1 $$r_{t}=\frac{M_{0}-M_{t}}{M_{0}}\times 100\text{\%},$$

where *M*_0_ is the initial mass of the slice and *M*_t_ mass at time *t*. It should be noted that an altered layer is formed to bond the surface of slices except QS–Na, but some of the altered layers could not be removed by washing with water. This surface layer could interfere with mass-loss measurements and the quantification of dissolution rates. To address this challenge, we removed the surface layer by dissolving it in a 4.0 mol l^−1^ HCl solution after the immersion experiment. SEM confirmed that submerging the slices in acid for 8 h completely removed the precipitated layer.

### 2.3. Ion concentration changes in reaction solution

In the first four systems, the reaction solution was collected by filtering after the quartz glass slices were taken out. Then, the ion concentrations in the filtrate including Li, Na and Si were analysed using an Optima7000DV inductively coupled plasma atomic emission spectrometer (ICP-AES) after diluting 2000 times by deionized water with 2% HNO_3_ to calibration. The concentration of Ca^2+^, which was dependent on the pH (or OH^−^ concentration) of the saturated hydroxide solution, was not measured.

### 2.4. Analysis of the reaction products

#### 2.4.1. Quartz glass powder immersion in different alkaline solutions

The quartz glass powder immersion experiment allows for the determination of the chemical process and the reaction products after immersion in different alkaline solutions at different temperatures. Quartz glass powder has small size fraction and large specific surface area and, therefore, is more reactive than quartz glass slices in alkaline solutions. The design of immersion experiments is summarized in table 2. All samples contained 2.0 g of quartz glass with or without Ca(OH)_2_. Two Ca(OH)_2_ to QP ratios were tested, including 0.1 : 2.0 g and 0.5 : 2.0 g. Samples were immersed in different alkaline solutions (each 50 ml). Samples were sealed in polypropylene copolymer containers and reacted at the preset temperature. The reaction time of most samples was 28 days, but the reaction rate of some samples was very low when the reaction temperature was 25°C, indicating that the reaction time may be extended appropriately.

**Table 2.** Design of quartz glass powder immersion experiment.

samples	quartz glass (g)	solution amount (ml)	Ca(OH)_2_ (g)	NaOH (mol l^−1^)	LiNO_3_ (mol l^−1^)
QP–Na	2.0	50	—	1.0	—
QP–Li	2.0	50	—	1.0	1.0, 2.0, 4.0
QP–0.1Ca	2.0	50	0.1	1.0	—
QP–Li–0.1Ca	2.0	50	0.1	1.0	1.0, 2.0, 4.0
QP–Li–0.5Ca	2.0	50	0.5	1.0	1.0, 2.0

#### 2.4.2. X-ray diffraction

Samples were taken up from the polypropylene copolymer containers when the reaction was over. Then, the products from all powder immersion experiments were filtrated by filter paper and washed three times by deionized water. The filtration products were oven-dried at 60°C for 24 h. Surface products of quartz glass slices of the fifth system were collected after rinsing by deionized water and oven-drying. It should be noted the vacuum drying oven was used to avoid carbonization when the sample contained calcium. Finally, changes in phase composition were analysed using the Rangaku Smart Lab XRD instrument, and data were collected at 30 kV from 2*θ* = 5° to 80° for the duration of about 10 min.

#### 2.4.3. Scanning electron microscopy/energy-dispersive spectroscopy

Type S-4800 scanning electron microscope (SEM) with energy-dispersive spectroscopy (EDS) was applied to observe the morphology of some resulting powder and the products on the surface glass slices of the fifth system in table 1. The upper surface and cross-section of every product on glass slice surface were observed.

## 3. Results

### 3.1. Mass-loss rate of quartz glass slices

**Figure 1.**
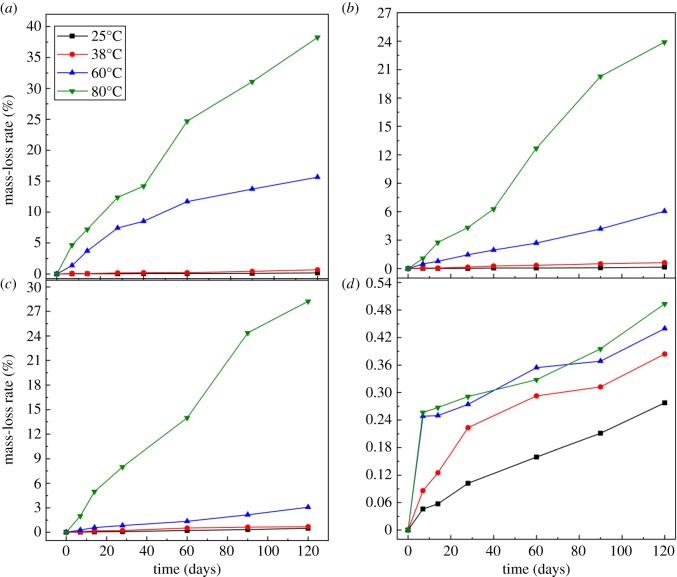
The mass-loss rate of quartz glass slices in different alkaline solutions. (*a*) QS–Na; (*b*) QS–Li; (*c*) QS–Ca; (*d*) QS–Li–Ca.

Figure 1 shows the mass-loss rate or the dissolution rate of quartz glass slices in four types of alkaline solutions (the first four systems). Each point in figure 1 represents a new quartz glass slice for periodical measurement. In the QS–Na system, the mass-loss rates were largely different between low and high temperatures (figure 1*a*); during the same period of 120 days, the mass-loss rates were less than 1.0% at 25 or 38°C but rose to 15% at 60°C and 40% at 80°C. These results suggest that the dissolution rate of quartz glass slice at low temperature was very slow even in pure NaOH solution, and more attention should be paid to the situation at high temperature. Figure 1*b*,*c* shows the similar change rule with time that the mass-loss rates were largely discrepant between low and high temperatures. In the case of 60 and 80°C, the erosion of OH^−^ to active SiO_2_ could not be stopped even in the solution containing Li^+^ or Ca^2+^. However, in terms of absolute value, the mass-loss rate of active SiO_2_ mitigated at high temperature when the solution contained Li^+^ or Ca^2+^. The change rule of mass-loss rate was completely different when the alkaline solution contained both Ca^2+^ and Li^+^ (figure 1*d*). First, the absolute value of mass-loss rate was much smaller than under other conditions and the differences were not very obvious among the four test temperatures, because all mass-loss rates were not larger than 0.5% even at 80°C. It should be noted that the mass-loss rates at 60 and 80°C sharply increased before 3 days and levelled off after 3 days, indicating that a reaction product may be rapidly generated on the glass surface to protect OH^−^-induced glass corrosion at high temperature, as elaborated in §3.3.

**Figure 2.**
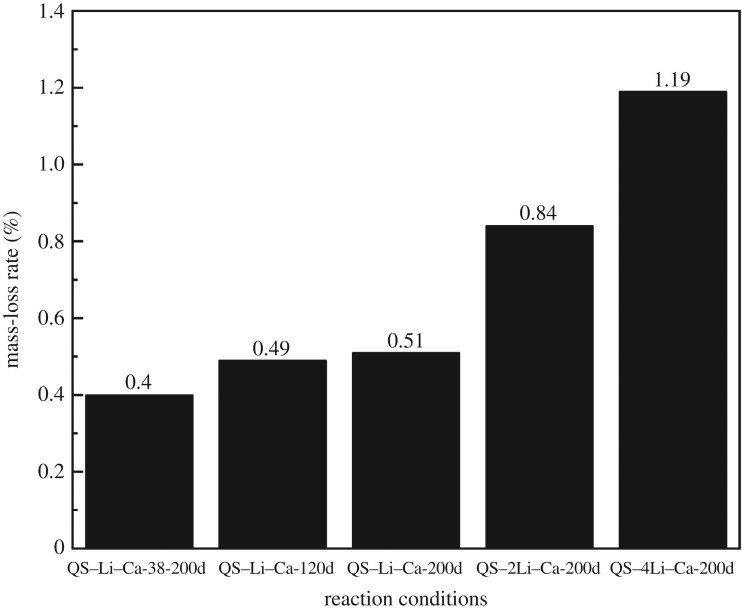
Effect of LiNO_3_ concentration on mass-loss rate of quartz glass slices at 120 or 200 days.

Figure 2 shows the effect of LiNO_3_ concentration on mass-loss rate of quartz glass slices at 120 or 200 days, and table 1 lists the detailed reaction conditions of every label in figure 2. Clearly, the mass-loss rate of QS was very stable and increased by 0.02% from 120 to 200 days with the presence of 1 mol l^−1^ LiNO_3_. However, with the increase of LiNO_3_ concentration from 2 to 4 mol l^−1^, the mass-loss rate also rose from 0.74 to 1.09% at 200 days. This result indicates too high LiNO_3_ concentration weakened the inhibiting effect on the OH^−^-induced quartz glass corrosion, but the mass-loss rates in all samples with different concentrations of LiNO_3_ (with Ca(OH)_2_) were much smaller than in other types of alkaline solutions.

### 3.2. Ion concentration changes

**Figure 3.**
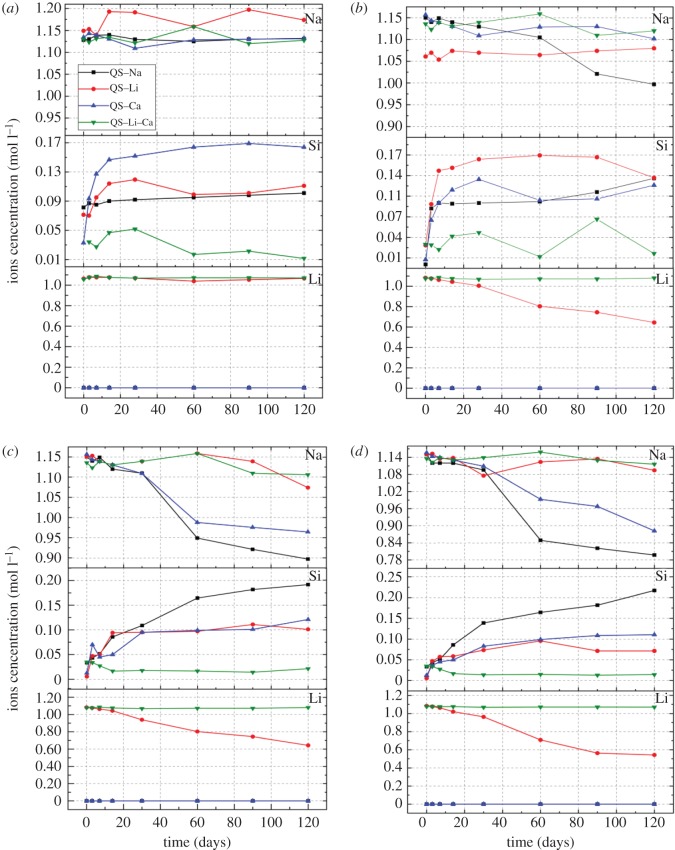
Ions concentration changes in different alkaline solutions at different temperatures: (*a*) 25°C, (*b*) 38°C, (*c*) 60°C, (*d*) 80°C.

Figure 3 shows ion concentration changes in four alkaline solutions at 25, 38, 60 and 80°C. Only the non-reacted ions or the balanced ions are shown, but not the ions combined in the products. Particularly, the Si ions resulted from the dissolution of quartz glass, but their initial concentrations were unknown. At 25 and 38°C, the concentration changes of different ions were almost similar and not obvious, as the maximum change was less than 0.2 mol l^−1^, which is consistent with figures 1 and 2. For Na^+^, the concentration in the 1 mol l^−1^ NaOH solution was almost unchanged at 25°C and declined slightly at 38°C, but dropped markedly at 60 and 80°C. These results indicate that an Na-bearing product was produced in this reaction system. The situation of Si ions was most complicated, because the Si ion concentration depended on two factors: the dissolution rate of quartz glass by OH^−^ and the association rate of Si ions with the product, and these two factors were affected by temperature, ion species and pH. However, at 25 and 38°C, the Si ion concentrations in all alkaline solutions changed the same way except in the QS–Li–Ca system. In other three alkaline solutions, the Si ion concentration increased at early stage (before 28 days) and then stabilized, indicating that the Si ions were under dynamic equilibrium between glass dissolution and product combination at later stage. The ion concentration changes indicate that Li^+^ combines other ions more easily than Na^+^, because the Na^+^ concentration remained nearly the same in Li-containing solutions but dropped sharply in Li-free solutions. Furthermore, the alkaline solution containing both Li^+^ and Ca^2+^ was the most stable, because the concentrations of all types of ions remained almost the same. However, it cannot be stated that the reaction system did not experience any change because the changes of ion concentrations would be wrong when the measurement method was ICP, especially in the solutions diluted 2000 times.

### 3.3. Reaction products

#### 3.3.1. XRD results

**Figure 4.**
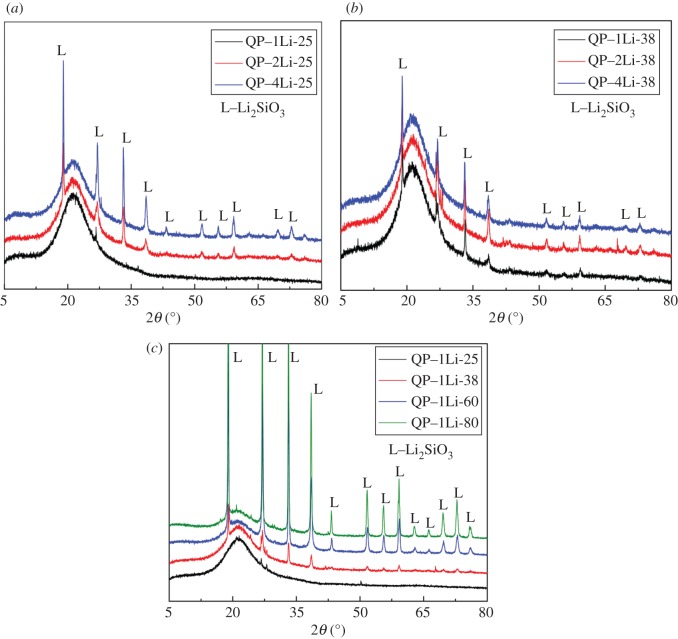
Effects of LiNO_3_ concentration and temperature on products about quartz glass powder in 1 mol l^−1^ NaOH solution. (*a*) Different concentrations of LiNO_3_ at 25°C after 50 days; (*b*) with different concentrations of LiNO_3_ at 38°C after 28 days and (*c*) with 1 mol l^−1^ LiNO_3_ at different temperatures.

To study the reaction products in quartz glass powder immersion experiments, we analysed all the samples in table 2 by using XRD. Figure 4 shows the effects of LiNO_3_ concentration and reaction temperature on products after reaction with quartz glass powder in 1 mol l^−1^ NaOH solution. Li_2_SiO_3_ was the only crystalline compound in most reaction conditions and a higher LiNO_3_ concentration led to a more intense diffraction peak of Li_2_SiO_3_ (figure 4*a*,*b*). However, when the LiNO_3_ concentration was 1 mol l^−1^ at 25°C, no crystal peak was found on the XRD patterns even when the reaction time was extended to 50 days. The XRD patterns of quartz glass powder in 1 mol l^−1^ NaOH solution with 1 mol l^−1^ LiNO_3_ show one crystalline characteristic peak (Li_2_SiO_3_) when above 25°C (figure 4*c*). Li_2_SiO_3_ is a low-solubility silicate, unlike the soluble Na_2_SiO_3_ or K_2_SiO_3_, so Li_2_SiO_3_ can exist stably in reaction solution other than the ASR gel or Na–Si gel with liquidity and instability. That is why the dissolution rate does not increase when the concentration of excess NaOH increases [22,25]; however, it accelerated Li_2_SiO_3_ generation with the increasing concentration of Li^+^.

**Figure 5.**
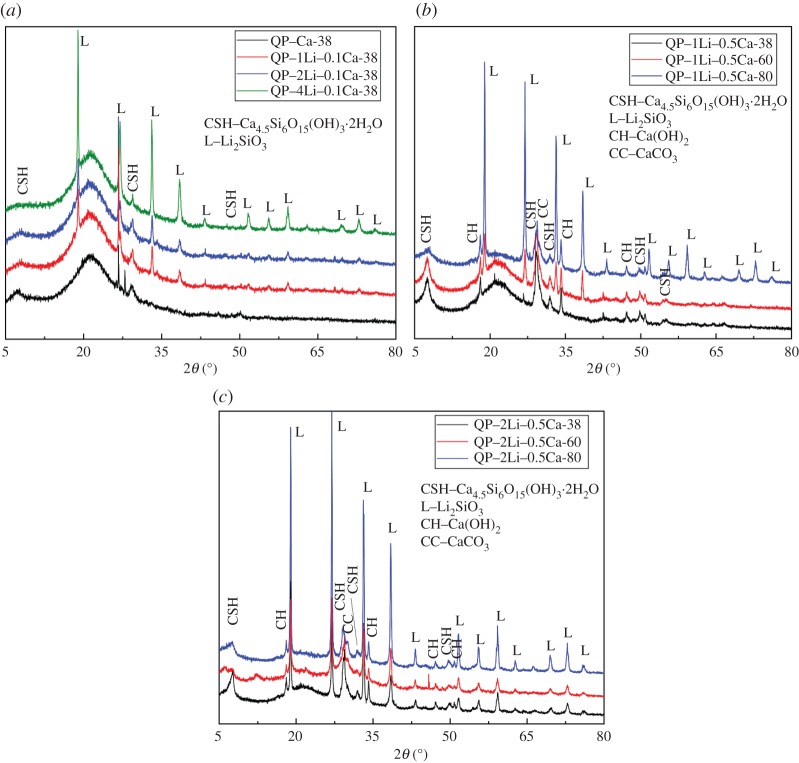
XRD patterns of quartz glass powder in 1 mol l^−1^ NaOH solution with LiNO_3_ and Ca(OH)_2_, (*a*) with different concentrations of LiNO_3_ and 0.1 g of Ca(OH)_2_; (*b*) with 1 mol l^−1^ LiNO_3_ and 0.5 g of Ca(OH)_2_ and (*c*) with 2 mol l^−1^ LiNO_3_ and 0.5 g of Ca(OH)_2_.

A large quantity of Ca(OH)_2_ was produced from Portland cement hydration, so the pore solution of concrete was saturated for Ca(OH)_2_. Therefore, Ca(OH)_2_ or Ca^2+^ was a main influence factor when studying the effect of Li^+^ on the process or product of ASR. Figure 4*a* shows the XRD patterns of quartz glass powder in 50 ml of 1 mol l^−1^ NaOH solution with 1 mol l^−1^ LiNO_3_ and 0.1 g Ca(OH)_2_ at 38°C and 28 days. First, characteristic peaks of Li_2_SiO_3_ crystals appeared in all XRD patterns as long as the alkaline solution contained 1 or 4 mol l^−1^ LiNO_3_. In other words, at 38°C, Li_2_SiO_3_ would be produced irrespective of Ca(OH)_2_. However, the largest difference between the Ca-containing system and Ca-free systems was the presence of other weaker peaks with broader full width at half maximum, leading to the very low crystallinity phase. This crystalline phase was found to be CSH [Ca_4.5_Si_6_O_15_(OH)_3_·2H_2_O] by referring to XRD PDF standard cards and literature [13,26]. So far, the structure and properties of CSH, or the difference between CSH and traditional C–S–H [27,28] in cement paste, are not fully understood. However, in a solution containing more Li^+^, the diffraction intensity of CSH is weaker (figure 5*a*).

There is no diffraction peak of Ca(OH)_2_ or CaCO_3_ (carbonization of Ca(OH)_2_) in figure 5*a*, indicating that Ca(OH)_2_ was almost consumed in the reactions. Therefore, it was unclear whether Li_2_SiO_3_ was produced after the complete consumption of Ca(OH)_2_ or simultaneously with the formation of CSH. To study the reaction sequence of Li_2_SiO_3_ and CSH, we repeated the quartz glass powder immersion experiments with 0.5 g of Ca(OH)_2_ added to the reacting system (50 ml). Figure 5*b*,*c* shows the XRD results after reactions for 28 days. The QP–Li system in figure 5*b* was somehow different from other systems at 38°C, because it produced only abundant CSH. The diffraction peaks of Li_2_SiO_3_ and CSH appeared only at 60 and 80°C. The peaks of Ca(OH)_2_ and CaCO_3_ were present at all temperatures even if a vacuum oven was used. The results in the solution of 1 mol l^−1^ NaOH with 2 mol l^−1^ LiNO_3_ (figure 5*c*) were similar to the solution with 1 mol l^−1^ LiNO_3_. However, diffraction peaks of Li_2_SiO_3_ were present at all temperatures and apparently more intense than in figure 5*b*. The dispersion peak of quartz glass was very weak in this situation, which indicates the high degree of reaction. Similarly, the diffraction peaks of Ca(OH)_2_ and CaCO_3_ existed at all temperatures. Even the systems with the unreacted Ca(OH)_2_ produced Li_2_SiO_3_ (figure 5*b*,*c*), indicating Li_2_SiO_3_ and CSH were produced simultaneously. Other information of concern in figure 5*b*,*c* was that the peak of quartz glass was weaker at higher LiNO_3_ concentration, indicating Li^+^ would accelerate the dissolution of quartz glass powder and promote the formation of Li_2_SiO_3_ in alkaline solutions. This conclusion proved the viewpoint wrong that Li^+^ can decelerate the dissolution of active aggregate in alkaline solution [2].

**Figure 6.**
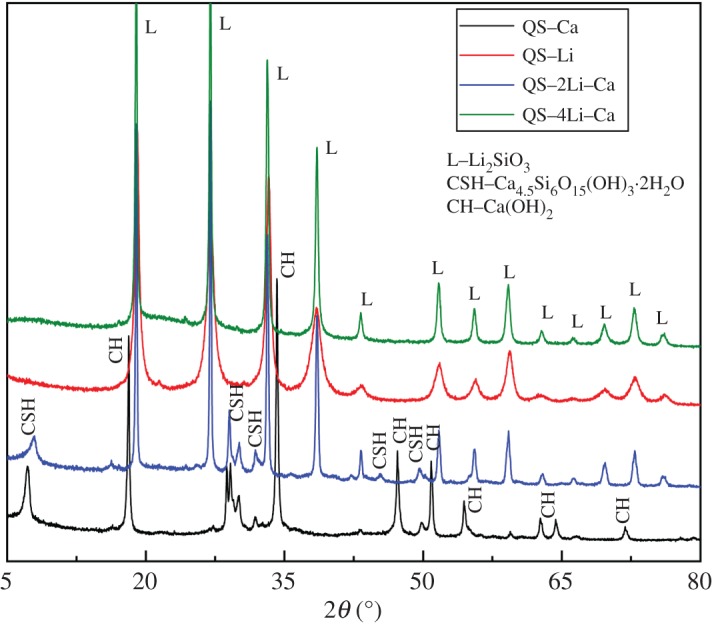
XRD patterns of products on surface of quartz glass slices in different alkaline solutions.

It should be noted that the production was very low in all alkali solutions in QS immersion experiment when at 25 or 38°C, because the reactions were very slow at low temperatures. Therefore, we only focus on the temperatures 60 and 80°C. Little product was generated in the 1.0 mol l^−1^ NaOH solution at high temperature, even though the mass-loss rate was very large. Unfortunately, only some flocculent product was formed, but cannot be further analysed because this product was an amorphous fluid, which should be ASR gel [22,29,30]. Figure 6 shows the XRD patterns of products after immersion in different alkaline solutions, if the products can be detected (XRD cannot detect products in QS–Li–Ca and QS–2Li–Ca-38). In QS–Li, the product was simplex Li_2_SiO_3_ and corresponded to the results of QP immersion experiments. The situation was complicated with the addition of Ca^2+^. When Li^+^ was absent, the product was CSH and the unreacted Ca(OH)_2_ can be detected by XRD (QS–Ca in figure 6). However, the ASR gel should be one of the products in this condition, because the Na^+^ concentration continuously declined (figure 3), as Na^+^ is one main component of the ASR gel, which is an amorphous fluid and thus cannot be detected by XRD. With the addition of both Li and Ca, the mass-loss rate was very small even at 80°C. Similarly, the product yield was also very small. The products of QS–2Li–Ca and QS–4Li–Ca can be detected by XRD, but XRD only shows the peak of quartz glass in other samples. The XRD patterns of QS–2Li–Ca showed that the products on the surface of QS were composed of CSH and Li_2_SiO_3_. These results are consistent with the quartz glass powder immersion experiments. However, as for QS–4Li–Ca, the XRD patterns only showed Li_2_SiO_3_, but not CSH. The possible reason was that the too high LiNO_3_ concentration led to the very small amount and relatively weak diffraction intensity of CSH. More details will be presented in §3.3.2.

#### 3.3.2. Scanning electron microscopy/energy-dispersive spectroscopy results

**Figure 7.**
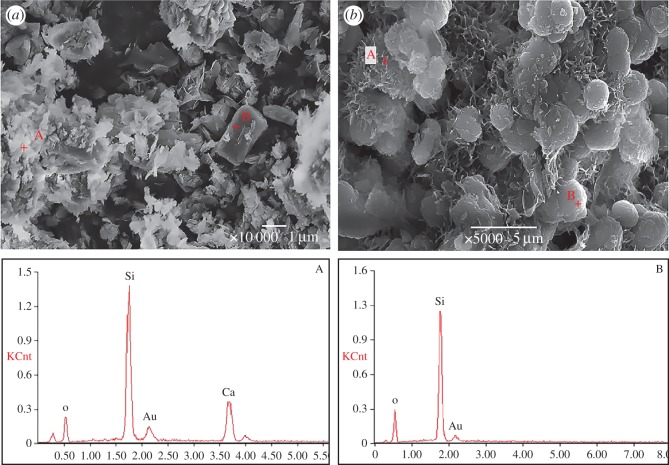
The SEM images and EDS data of quartz glass powder in 1 mol l^−1^ NaOH solution with Ca(OH)_2_ at 80°C, (*a*) with 1 mol l^−1^ LiNO_3_; (*b*) with 2 mol l^−1^ LiNO_3_.

Figure 7 shows the SEM images and EDS data of quartz glass powder in QP–1Li–Ca and QP–2Li–Ca at 80°CC, and figure 5 shows the phase compositions of reaction products. As mentioned before, the XRD patterns in figure 5 indicate that the products mainly consist of CSH and Li_2_SiO_3_. The SEM images show two types of morphology or phase composition: the irregularly shaped and flocculent or meshy products (indicated by A) and the regularly shaped and bulk or ball shape products (indicated by B). The EDS data show that product A containing Ca, Si and O should be CSH, and product B containing Si and O should be Li_2_SiO_3_ because the quartz glass (SiO_2_) was almost completely consumed and Li cannot be detected by EDS. A higher Li^+^ concentration facilitated the generation and crystallization of Li_2_SiO_3_, and the CSH may act as a bridging and filling agent in the gaps between Li_2_SiO_3_ crystals, making the product more compact.

**Figure 8.**
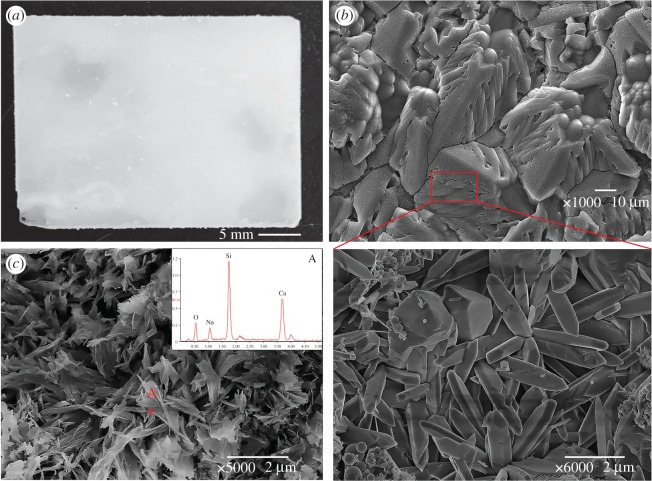
Reaction products of quartz glass slice immersion in alkali solutions at 200 days. (*a*) Photo of QS–Li; (*b*) SEM images of QS–Li and (*c*) SEM image of QS–Ca and its data of EDS.

After the quartz glass slices were immersed in the QS–1Li solution for 200 days, the glass surface was completely encased by Li_2_SiO_3_ (white matter in figure 8*a*). However, the combination between Li_2_SiO_3_ and glass was incompact and the Li_2_SiO_3_ crystals easily dropped off the glass surface. SEM shows that the crystalline grains grew well, but contained many inter-grain gaps, which became the channels for ion migration. In other words, this product layer cannot protect against the glass corrosion by OH^−^. These results accord with the data of mass-loss rate. The product of quartz glass slice immersion in QS–Ca consisted of Na, Ca, O and Si (figure 8*c*), indicating that the product may be CSH and ASR gel. Regardless of the composition, SEM shows that this product is very loose and cannot bind tightly to the glass surface, and thus cannot be the protective layer against the glass corrosion by OH^−^. This conclusion is also consistent with the results of mass-loss rate.

**Figure 9.**
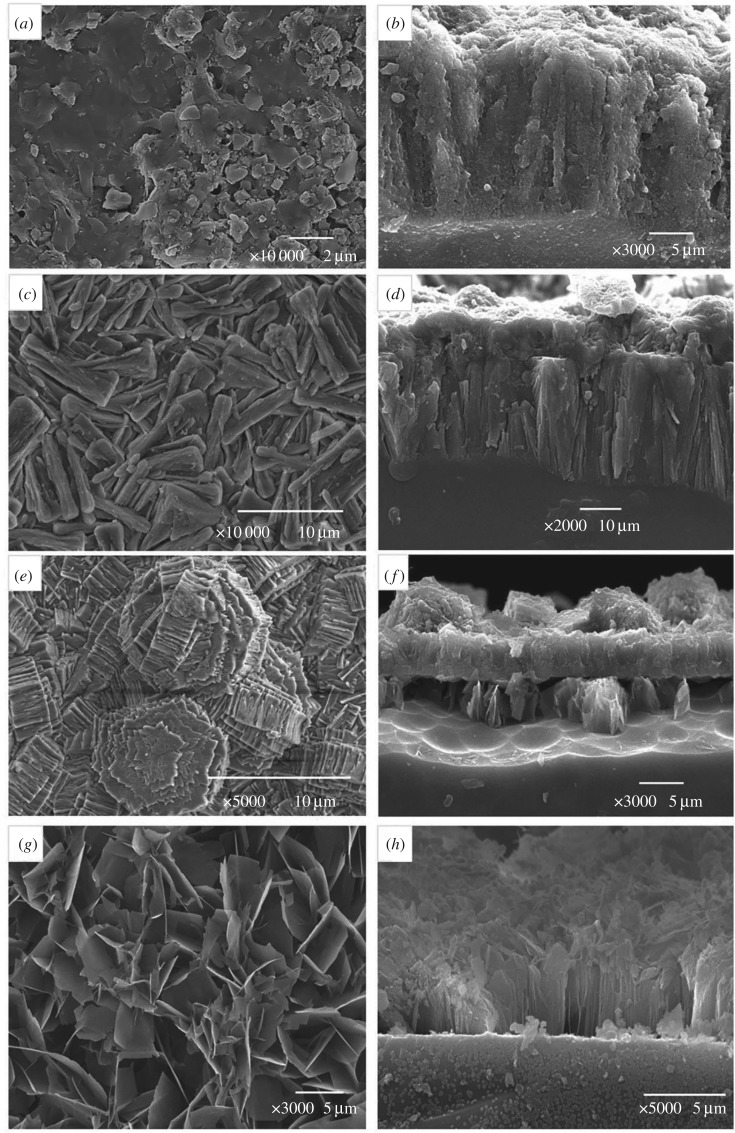
SEM images of product layer at different conditions. (*a*) Upper surface and (*b*) cross-section of QS–Li–Ca; (*c*) upper surface and (*d*) cross-section of QS–2Li–Ca; (*e*) upper surface and (*f*) cross-section of QS–4Li–Ca; (*g*) upper surface and (*h*) cross-section of QS–2Li–Ca-38.

Figure 9*a*–*h* shows the SEM images of products on the surface of quartz glass slices at different conditions (system 5). Each product was observed on both upper surface and cross-section. When the LiNO_3_ concentration was 1 mol l^−1^, the product cannot be detected by XRD, but a compact product layer about 15 µm thick was formed on the glass surface when at 80°C after 200 days (figure 9*a*,*b*). This product layer was very dense and bound tightly to the surface of quartz glass and can prevent OH^−^ from corroding quartz glass. When the LiNO_3_ concentration was 2 mol l^−1^, SEM showed that numerous rod-like crystals were produced on the glass surface and the cross-section SEM image showed a product layer about 25 µm thick appeared on the surface (figure 9*c*,*d*). This layer was constituted by many longitudinally arranged rod-like crystals, but the compact degree was less than the layer formed in the 1 mol l^−1^ LiNO_3_ solution. When the LiNO_3_ concentration further rose to 4 mol l^−1^, the XRD patterns only showed the peaks of Li_2_SiO_3_, but not CSH. Figure 9*e*,*f* shows the upper surface and cross-section SEM images of glass slice, respectively, in the 4 mol l^−1^ LiNO_3_ solution at 80°C for 200 days. Many rod-like crystals aggregated on the surface of the product layer. However, the cross-section SEM image indicated that this product layer was divided into a plate-like loose structure and a dense massive structure from down to up. EDS shows that the loose layer containing Ca, Si and O may be CSH and cannot be detected by XRD, because it is covered by the dense layer which has no Ca. SEM images indicate that this product layer was not closely tied to the surface and just like the alkaline solution with Li but without Ca, which explains why the mass-loss rate of QS increased at too high Li^+^ concentration. Figure 9*g*,*h* show the surface and cross-section SEM images of the product layer in QS–2Li–Ca-38 after 200 days. A product layer less than 5 µm thick appeared on the slice surface, as the surface of the product layer was covered by a plate-like structure and the cross-section was composed of a longitudinally arranged rod-like structure. This result indicates that the product layer also can be formed at low temperature.

## 4. Discussion

The quartz glass powder immersion experiment shows that Li_2_SiO_3_ is the only Li-bearing product, which is not affected by the temperature, Li^+^ concentration or addition of Ca. The higher reaction temperature and higher Li^+^ concentration accelerate the formation of Li_2_SiO_3_. Figure 5 shows that Ca^2+^ combines silicate ions regardless of Li^+^. The reaction system without Li^+^ produces CSH, which can be detected by XRD and ASR gel [30,31], which can be indirectly confirmed by ion concentration changes (figure 3) and EDS (figure 7). If the solution contains Li^+^, the quantity of CSH decreases significantly with the increase of Li^+^ concentration and does not produce ASR gel. Moreover, the CSH and Li_2_SiO_3_ would be produced simultaneously or coexist (figures 3 and 7). The formation mechanism of Li_2_SiO_3_ crystal can be described as follows.

When the quartz glass is immersed in alkaline solution (NaOH solution), the (≡Si–O^−^) bonds will progressively be attacked by the hydroxyl (OH^–^) ions, resulting in network dissolution of silica [29]:

4.1 $${\left(\equiv \text{Si}-\text{OH}\right)}_{s}+3{\left(\text{O}{\text{H}}^{-}\right)}_{\mathrm{aq}}↔{\left(\text{SiO}{\text{H}}_{4}\right)}_{\mathrm{aq}},$$

where (≡Si–OH) represents the silanol groups that are present at the silica–water interface. If the alkaline solution does not have Li^+^ and Ca^2+^, the Na ion exchange reactions will occur:

4.2 $${\left(\text{SiO}{\text{H}}_{4}\right)}_{\mathrm{aq}}+\text{N}{\text{a}}_{\mathrm{aq}}^{+}↔{\left((\text{H}{\text{O}}_{3}\equiv \text{Si}-{\text{O}}^{-})\ldots \text{Na}\right)}_{\mathrm{aq}}+{\left({\text{H}}^{+}\right)}_{\mathrm{aq}},$$

where O^−^ … Na^+^ indicates that the bond is more likely a strong van der Waals type. With the increase of the concentration about ((HO_3_≡Si–O^−^) … Na)_aq_, a kind of ASR gel will be generated in solutions. If the alkaline solution contains Ca^2+^, Ca^2+^ can link silica ions to form poly-metalsilicates:

4.3 $$2{\left({\left(\text{HO}\right)}_{3}\equiv \text{Si}-{\text{O}}^{-}\right)}_{\mathrm{aq}}+{\left(\text{C}{\text{a}}^{2+}\right)}_{\mathrm{aq}}↔{\left(\text{H}{\text{O}}_{3}\equiv \text{Si}-\text{O}\ldots \text{Ca}\ldots \text{O}-\text{Si}\equiv \text{O}{\text{H}}_{3}\right)}_{\mathrm{sol}}.$$

With the increasing concentration about (HO_3_≡Si–O … Ca … O–Si≡OH_3_)_sol_, CSH will be generated in solutions. However, if the alkaline solution contains Li^+^, because the Li_2_SiO_3_ with very small solubility and has the good chemical stability, Na^+^ does not chance to occur ion exchange reactions, and the following precipitation reaction will occur:

4.4 $${\left({\left(\text{HO}\right)}_{3}\equiv \text{Si}-{\text{O}}^{-}\right)}_{\mathrm{aq}}+2{\left(\text{L}{\text{i}}^{+}\right)}_{\mathrm{aq}}\to (\text{L}{\text{i}}_{2}\text{Si}{\text{O}}_{3})\text{s}+{\left(\text{O}{\text{H}}^{-}\right)}_{\mathrm{aq}}.$$

Therefore, the reaction system will not generate ASR gel. But the Ca^2+^ can link oligomer silicate and the CSH will also be generated even if the solution contains Li^+^.

The quartz glass powder immersion experiment shows that the CSH and Li_2_SiO_3_ are the only two products in the system containing Li^+^ and Ca^2+^. Thus, the effects of Li^+^ and Ca^2+^ on the dissolution of quartz glass slices by OH^−^ can only be attributed to CSH and Li_2_SiO_3_. The mass-loss rates of quartz glass slices in different alkali solutions indicate that the corrosion of quartz glass can be effectively inhibited only when the alkali solution contains both Li and Ca, regardless of temperature, Li^+^ concentration or reaction time. SEM uncovers a type of product layers on the surface of quartz slices, and the layer thickness, structure and morphology depend on the temperature and Li^+^ concentration. However, such product layers can effectively prevent OH^−^ from eroding quartz glass. XRD and SEM/EDS confirm that the product layers are constituted by Li_2_SiO_3_ and CSH, but with largely different proportions among product layers. However, too high LiNO_3_ concentration influences the compactness and inhibition effectiveness of the product layer because CSH can be hardly formed at high LiNO_3_ concentration and CSH plays the role of a binder filler in the product layer.

In summary, the reason of low mass-loss rate in Li-containing alkaline solutions is that Li and Ca can produce a compact product layer on the glass surface, which is constituted by Li_2_SiO_3_ and CSH and acts as a physical protective layer against the migration of OH^−^ and alkali ions to the glass surface. Ca^2+^ plays two very important roles in this protective layer. Firstly, Ca^2+^ can hinder the combination between silicate and lithium ions to form lithium silicate and inhibit the crystal growth of lithium silicate with a columnar close packing structure. Secondly, Ca and silicate can form CSH, which makes the product layer combine better with glass surface, and fills the lithium silicate crystal gaps, making the product layer denser and less porous.

## 5. Conclusion

1. Mass-loss rates of quartz glass slices were less than 1% at 120 days at low temperatures (25 and 38°C) in all alkali solutions. At high temperature (60 and 80°C), mass-loss rates of quartz glass in NaOH solution, alkaline solution only with Ca and alkaline solution only with Li were more than 20% for 120 days. The mass-loss rates in the alkaline solution with both Ca and Li were lower than 0.5% for 120 days or longer at all temperatures, but too high LiNO_3_ concentration increased the mass-loss rate.
2. Li_2_SiO_3_ crystals were the only product when the quartz glass was immersed in alkaline solutions only with Li, and the formation rate related to temperature and lithium ion concentration. CSH was produced when the alkaline solution contained both Li and Ca; the existence of Ca or CSH influenced the growth of Li_2_SiO_3_ crystals and reduced the crystal size.
3. The quartz glass slices were almost not corroded by OH^−^ in alkaline solution containing both Li and Ca, because a type of production layer was formed on the glass surface, which was mainly composed of CSH and Li_2_SiO_3_ crystals and had a dense structure and interface bonding. The product layer can stop OH^−^ from migrating to the surface to corrode the quartz glass.

According to the results, the reaction products and the effect of Li^+^ on ASR were presented when quartz glass was used as reactive aggregate. However, the situation concerning the effect of Li^+^ on ASR in the concrete with kinds of natural reactive aggregates is more complicated and variable, which should be further studied in the future.

## Data accessibility

All data are included in the manuscript.
